# The meaning of early intervention: A parent's experience and reflection on interactions with professionals using a phenomenological ethnographic approach

**DOI:** 10.3402/qhw.v10.25891

**Published:** 2015-07-21

**Authors:** Yoon H. Lee

**Affiliations:** Department of Psychological, Organizational, Leadership Studies in Education, College of Education, Temple University, Philadelphia, PA, USA

**Keywords:** Phenomenological ethnographic approach, family-professional partnership, family-centered practice, parent participation, early intervention

## Abstract

The purpose of this study is to describe how a parent's partnership with professionals progresses and evolves throughout the service provisioning process. Using a phenomenological ethnographic approach, the lived reality of a family is depicted as the parent walks through different stages of the Individualized Family Service Plan process over a 6-month period. Data concerning parent–professional interactions were obtained via observation notes and document reviews whereas data regarding parent perceptions were collected through multiple individual interviews. Overall, the parent conveyed her satisfaction with actual services especially regarding the professionals’ knowledge and parental advocacy. However, the parent also indicated frustration with the early intervention planning process and “obligated” partnerships with providers. In particular, the providers’ lack of sensitivity was noted, and greater emotional and psychological support was suggested. The overall process of developing partnerships with professionals can be excessively intrusive to the family's lives. Future research directions are offered as a contribution for the development of improved policies for early intervention programs regarding family-centered practice, utilizing the perspectives of families.

The parent–professional partnership is a well-established ontological framework in early intervention^1^ in the United States, Australia, China, Germany, Ireland, and many other nations (James & Chard, 2010; Odom & Kaul, 2003). The field of early intervention has focused on close, quality interactions between families and professionals in the service planning and delivery process (Brotherson, Cook, Erwin, & Weigel, 2008; Bruder & Dunst, 2015; Coogle & Hanline, 2014; Johnston, 2003; Summers, Hoffman, Marquis, Turnbull, & Poston, 2005; Turnbull et al., 2007; Wehman & Gilkerson, 1999; Ziviani, Darlington, Feeney, Rodger, & Watter, 2013; Ziviani, Feeney, & Khan, 2011). Assisted by professionals, families are encouraged to make decisions and choices about services for their children in light of their resources and priorities in their everyday lives. Because the child spends most of his or her time within the family, families are viewed as experts in understanding the child's disability and the family's needs. Within this framework, a crucial element of service planning and delivery has been in the development of an Individualized Family Service Plan (IFSP), which is established within the family–professional partnership (Bailey & McWilliam, 1993; Bailey, Raspa, & Fox, 2012; Jung, 2010; McWilliam et al., 1998; Turnbull et al., 2007).

## Significance of collaborative interactions in early intervention

Since its inception in the 1980s, and throughout the law's multiple reauthorizations in the United States, early intervention has served young children with disabilities and their families. Australia also established an inclusive early intervention service system, a couple of decades ago and adopted a family-centered approach as a recommended practice (Johnston, 2003; Sukkar, 2013). In an attempt to deliver family-centered practices in early intervention, the service planning and delivery process included the development of an IFSP to be established jointly by families and early intervention professionals (Bailey & McWilliam, 1993; Jung, 2010; McWilliam et al., 1998; Turnbull et al., 2007). In Ireland, for example, a comprehensive plan, including formulating an IFSP, is considered as a means for establishing an interdisciplinary model and collaboration in early intervention (Carroll, Murphy, & Sixsmith, 2013).

The individualized plans incorporate families as effective, active team members. Early intervention professionals must encourage families’ active participation; address their concerns, needs, and strengths; and prioritize their focus by choosing desirable settings and learning opportunities for early intervention services (Bailey, Scarborough, Hebbeler, Spiker, & Mallik, 2004; Gallagher, Rhodes, & Darling, 2004). In addition to the therapies and special education services that the child receives, additional family support services, such as home visits, counseling, resources, and educational activities, must be considered for strengthening the family unit (Epley et al., 2010; Woods, Wilcox, Friedman, & Murch, 2011). By means of family-centered practices and the support of professionals, children and families are able to build the necessary competencies in their lives through participatory decision making and family support service provisions. Hence, the completion of the IFSP process denotes that professionals and families work collaboratively (Bailey & McWilliam, 1993; Turnbull et al., 2007; Zhang, Fowler, & Bennett, 2004; Ziviani et al., 2013).

## Understanding partnerships through multiple perspectives

Although the initial intention of early intervention was to provide individualized services for children with special needs and their families, in practice, professionals tend to lean toward a more professionally driven and less family-centered service model (Dunst, 2012; Jung & Grisham-Brown, 2006; Lee, 2015). Dunst particularly criticized how the practice relies on private providers and their services, referring to the field as an early intervention industry. In general, professionals meet and interact with families by sharing information about their child, such as his or her present level of development based on professionals’ evaluations. Thus, family–professional partnerships tend to develop from the viewpoint of professionals rather than that of families (Dunst, 2012). Parents sometimes position themselves as individuals with less expertise who require assistance from experts to increase their knowledge level and to aid the progress of their child's development and education. As a result, the relationship between parents and professionals does not always develop into the mutual relationship as described by the law, but tends toward a one-sided dependence of one group (i.e., parents) on another group (i.e., professionals) with greater knowledge and expertise (Lee, 2015).

This might be because the law, especially in the United States, is written from the perspectives of policy makers and service providers (Florian, 1995; Harry, 1992; Jung & Grisham-Brown, 2006; Valle & Aponte, 2002). The system of early intervention has been concerned with what early intervention can do for families, such as how families would collaborate with specialists through the demonstration of partnerships. Less is known about how families view relationships with professionals, and how partnerships evolve from the perspectives of families in the process of service planning and delivery.

The aims of the present study were to add another layer to the discourse on family–professional partnerships from a service user perspective. Considering the current issues vis-à-vis the partnership between families and professionals in early intervention, the purpose of the study is to portray the nature and the quality of parent–professional partnership in the service process by examining a parent's experiences and her reflections on the interactions. A parent's experience and perception are studied using a phenomenological ethnographic approach as she walks through the different stages of the IFSP process in the United States over a 6-month period.

Because the human world is a social product that can be subjectively interpreted by the multiple individuals, more “genuine understanding of the other person” should be sought (Schutz, 1967, p. xxv). Thus, the lived and the experienced reality of families, and, in particular, how their partnership with professionals evolves throughout the process of service provision, are investigated using a phenomenological ethnographic approach. The study addresses the following research questions: 1) How did the family interact with their service providers and professionals in the early intervention process, including referral, evaluation, IFSP meeting, and service delivery? 2) How did the family reflect on the partnership with early intervention professionals and service providers?

As an educated, middle-class researcher of Asian background, I have been involved in and participated in providing early intervention services in the United States for the past 13 years in various capacities. I have been a classroom teacher, early intervention evaluator, and am currently a teacher educator for varying special educational models. I have also been an observer, service provider, and parent advocate while working with diverse families. Being actively involved in the field and seeing how an uneven relationship between families and their service providers can occur in practice led me to this study. My identities influenced how I initially approached the participant and how I interacted with her throughout the study.

Given that this study focused on hearing family accounts while going through the process of accessing, planning, and receiving early intervention services, I opted to create a dialogical relationship between the participant and myself as a researcher (Blaise, 2005). As such, the parent and I discussed and shared our reflections throughout and at the end of each interview in addition to answering planned questions. Sharing reflections with the families was valuable in becoming aware of the parent's experience while working with professionals and in communicating any concerns in the data collection process. In addition, the collected data were also shared with the parent participant for her verification (i.e., member checks; see the “Data collection” section). Even though I have been a parent advocate, service provider, etc., my identity in the process of data collection was predominantly as a researcher. Therefore, it is possible that I, too, held a position with power, such as a researcher with an advanced knowledge base concerning service models, laws, and regulations of early intervention.

## Method

### Phenomenological ethnography

In an effort to investigate the differences of some aspects of lived experiences, the present study used a phenomenological ethnographic approach. Phenomenological ethnographic research enables us to “observe, describe, and classify the social world as clearly as possible in well-ordered terms” (Schutz, 1971, cited in Maso, 2001, p. 137; see also Merleau-Ponty, 1945/2012). Phenomenology is to seek insight into subjective experiences behind their perceptible reflections (Maso, 2001), whereas ethnography emphasizes a researcher's immersing engagement in the lives of others’ naturally occurring social situations (Atkinson, Coffey, Delamont, Lofland, & Lofland, 2001). Because parent–professional partnerships involve multiple interactions with others through the interpersonal dynamics of service planning and delivery, this approach serves the study's intent well. Phenomenological ethnographers take part in a process called “epoche” as suggested by Husserl, that is, being both an insider and an outsider in social situations, which allows critical analytic questioning and understanding of those being researched (Butt, 2004). Through active participation and reflexive observation, while deeply immersed in interactions with professionals, my insight into the lived and experienced meaning of parent–professional partnerships would be broadened (Larsen, 2007; Schutz, 1967).

In considering parent–professional partnerships, the study enables parents, professionals, and/or researchers to learn aspects of the lived experiences of parent–professional partnerships rather than “identify” individual differences in the partnerships or make any generalized claims (Ayres, Kavanaugh, & Knafl, 2003). This collection of information should illuminate “a more extensive, clearer, and more accessible idea of that phenomenon” of parent–professional partnerships (Maso, 2001, p. 143). The findings can be added to our collective ideas regarding what parent–professional partnerships encompass or scaffold the idea through transformative meaning (Csordas, 1994; Maso, 2001). By portraying the perspective of the service user, the results may lead to better understand the experience's meaning in the process of early intervention service (Schutz, 1967).

### The participant: a snapshot of Lily and her family

In the attempt of preserving the richness of individual experience by using a phenomenological ethnographic approach, the study attended to one individual's intensification (i.e., the use of a narrative that yields phenomenological meanings) and evocation (i.e., bringing experience vividly into presence; Ayres et al., 2003; Van Manen, 1997). Thus, the study focused on one family's experience of early intervention. Lily was a 34-year-old mother of two boys, 3-year-old HP, and 4-month-old Malcolm, at the time of recruitment. As part of a white, middle-class, and educated family, Lily (with a professional background in social work), her husband, and their two children lived in a northeastern urban city in the United States. At the time of the recruitment, 4-month-old Malcolm had been referred to early intervention. During the first week after his birth, Malcolm had a stroke for unknown reasons. The doctors in the pediatric neurology department of the hospital where Malcolm was an inpatient informed the family that they were not sure if he would walk or be able to do anything but what he was doing at that moment. The doctors subsequently suggested an early intervention referral.

Lily's interests included baking, blogging, and running. She once shared a website address with me of Malcolm's blog that she had created. There, she shared Malcolm's happenings, photos, medical treatments, progress, etc. A notable posting^2^ that I am compelled to share is:

I took Malcolm grocery shopping this afternoon and he was wearing his daytime hand splints. I noticed that people were looking at us and I thought, “I'm that woman now, with the child who is different/special/disabled.” I used to notice women like me and wonder what was wrong with their children. I would feel sorry for them, and thankful that HP [Malcolm's older brother] was (is) so healthy, strong, capable, [and] *normal*. Once when I was about 4 or 5 months pregnant with Malcolm, I was pushing HP in a swing. There was a mom beside us who was pushing her severely disabled child in one of those big sled-shaped swings. He was probably 7 or 8 years old. The image of her lifting his body out of his wheelchair and into the swing has stayed so close to me for the past year. It was my interpretation of the worst case scenario when Malcolm had the stroke and was in the pediatric ICU. Now I know the worst case scenario would have been if we lost him. But he is here now, sleeping soundly on my lap in his room at home. He was laughing an hour ago, when I was tossing him up in the air. Things could be much, much worse.

The above excerpt showed how Lily as a parent with a child with a disability came to negotiate with herself and her new world. In addition to my engagement with her and world, reading her blog helped me better understand what Lily as a parent of a child with special needs contended with in life.

Throughout the study, my relationship with the family exclusively involved Lily, who was the child's primary caregiver, for the purpose of grasping the meaning of her experiences (Schutz, 1967). Lily and I maintained a cordial relationship mostly via face-to-face meetings and emails. Because Lily was frequently interrupted by Malcolm during the interviews, we decided to try electronic communication via email after the first few interviews. When there were unanswered questions during the interview, a list of questions was sent and Lily replied back by email. This method of interaction worked well for both of us. As such, the study was able to obtain extensive data via email communication in addition to our in-person interview dialogues.

### Data collection^3^

Data concerning parent–professional interactions were obtained via observation notes and document reviews whereas data regarding parent perceptions were collected through multiple individual transcribed interviews. The overall data collection and analysis process followed the stages of early intervention service planning, including referral, evaluation, the IFSP meeting, and service delivery. When Lily came across my participation recruitment posting on a parent group website, her evaluation process had been completed and an initial IFSP meeting was scheduled the following week. Thus, my observations concerned the service planning and delivery process including the first IFSP meeting and early intervention service sessions. The interview series included an initial interview, follow-up interviews after each service planning and delivery session, and a final interview with the family. Finally, Malcolm's IFSP document was examined to see the ways that professionals communicated and interacted with the family (Table I). Three methods were triangulated in this study (Bogdan & Biklen, 2006; Stake, 2000) to capture and “concrete” subjective meaning of the individual's lived experience as a phenomenon (Denzin & Lincoln, 2000; Schutz, 1967).

**Table I:** Data collection process.

February	3	After viewing the posting on a parent group website, Lily emailed concerning her interest.
	11	The first meeting between Lily and the researcher took place at her house for about an hour. They introduced themselves, consent was signed, and the study's process was discussed. Lily talked about her early intervention experience, including referral and evaluation process thus far, as well as her professional background. She also asked about disclosure of the study's findings with the family.
	13	An IFSP meeting was held in the regional office of the family's neighborhood. Lily, Malcolm, the Early Intervention Official Designee (EIOD), an evaluation representative, a service coordinator, and the researcher participated. The purpose of the meeting observation was accounted for and occurrences and dialogues at the meeting were digitally recorded along with making observational notes.
	22	A follow-up interview after the initial IFSP meeting took place at the family's home.
March	21	A physical therapy session was observed at the family's home involving Lily, Malcolm, the therapist, and the researcher. After the therapy session, a follow-up interview was conducted.
May	5	A special instruction session was observed at home involving Lily, Malcolm, the therapist, the family's child-care provider, and the researcher. A follow-up interview was conducted in person and via emails.
June	11	Checking-in and continued conversations were conducted via emails. Lily answered some follow-up questions regarding the IFSP meeting and early intervention services and sent the IFSP document via email.
August	13	Continued communication via emails. Again, Lily answered follow-up questions regarding the overall early intervention process and sent the IFSP document via email.

The analysis of collected data occurred in phases (Ayres et al., 2003; Bogdan & Biklen, 2006; Miles & Huberman, 2013). First, my approach was to immerse myself in the data by reviewing observation notes and transcripts to acquire a feeling for events featuring parent–professional interactions. Second, a collection of significant events from observations and notable statements from transcripts that had a direct relation to the interactions of the parent and professionals were identified. Third, categories for these events and statements that had commonalities were established. Fourth, returning to the data occurred to validate the categories by reconnecting each event and statement to the original context. Next came engaging in critical reflections on themes within the categories and free writing on those themes. In the end, a framework based on the common themes was established while pondering the study's research questions. Lily received a copy of my writing for evaluation to ensure member checks; no significant changes were made (Lather, 1986). Trustworthiness was considered through member checking and data triangulation (Merriam, 2009).

My aim in the following section is to describe my observations coupled with interview data on Lily's reflections on what was observed. In my observation notes, I attempt to holistically document occurrences and their context (Ayres et al., 2003; Stake, 2000). As a qualitative study, however, the researcher may have zoomed in on events that were interesting to her. “All material must be treated with equal horizontal importance as we try to capture how things appear to the interviewee” (Butt, 2004, p. 92). Thus, by tying my observations to interview data, I tried to capture the meaning of Lily's experience of her participation and interactions in the early intervention processes.

## Results: parent's early intervention story

Lily expressed satisfaction with the overall process of early intervention. However, elements of dissatisfaction included relationships in the planning process, decision making throughout the process of provisioning services, and burden of the family's responsibility to participate.

Upon entering into the system of early intervention, Lily had not known or heard about early intervention but was forewarned by a friend who had gone through the process that the process could be drawn out and frustrating. Because of her low expectations about early intervention and its processes, Lily stated that overall “it's been less painstaking” and “things have gone according to the timeline.” Regarding the parent–professional partnership, Lily especially found that the professionals’ knowledge helped her interact and develop the family's competencies and skills. She was especially satisfied with the physical therapist and her services because the therapist had specific skills and knowledge. Lily added that the physical therapist's advocacy aided the two's interactions. Because the professional was “knowledgeable about what our rights are [and] what the possibilities are,” Lily stated that this information had been helpful in navigating the early intervention system. For the most part, however, Lily did not care much about what happened or how things went throughout the provisioning process, but cared more about whether her family was able to secure the necessary services for Malcolm and how successfully those services were executed for the child.

### Relationships in the planning process

Especially in the evaluation process and at the first IFSP meeting, the parent recalled that she and the professionals seldom developed collaborative partnerships, and therefore their interactions were a mere formality. According to Lily, after the referral, various evaluators came to the family's home to observe and examine what Malcolm was capable of doing. In a series of visits, each evaluator asked Lily a series of questions that were simply verifying information or repeating questions. Lily recalled, for example, the evaluators asked “[if] I had any concerns [or] what were my hopes or thoughts or concerns about his conditions.” And Lily remembered stating, “I was anxious that he'd never walk, play, [or] do things that normal kids do [and] I was afraid he's going to be a vegetable or something like that.” However, the parent's concerns were not answered or dealt with but simply recorded. Lily reflected, “They just kind of nodded and wrote it down [Lily chuckles], so it was just kind of information gathering.” Lily's comments implied that there were few dialogues in these interactions.

In addition, throughout the IFSP meeting, dialogues between Lily and other participants were a formalized interaction, such as asking and answering routine questions. For example, the official, who led and managed the meeting, elucidated many questions regarding Malcolm's developmental performance, parental concerns, outcomes and goals, etc. from her list, such as “Tell me about things that he is doing right now developmentally,” “What have you noticed he is doing now, or you think he should be doing and he isn't at this point of time developmentally speaking?”, “Is there anything else that concerns you at this point in time?”, and “Based on the concerns you feel, what kind of things do you want him to do in the next few months?” To these questions, Lily simply shared information about Malcolm's development and the needs of the family; her responses were simply documented by the official and the service coordinator while no further discussion was made concerning the answers provided by the parent.

There were also incidents when the interactions were affected by the position of the official. When notifying the parent of the type of services and their frequency, the official seemed to maintain her authoritative position. According to the field notes:

The official began to list the recommendations, “Mrs. P [Lily's last name] … the physical therapy including the family training component, we are going to recommend three times a week for 30-minute sessions in your home, meaning there's going to be two sessions that are going to be focusing on him and you and there's going to be a session that's going to be focusing on you and him …. Family training should let you be able to practice when she's [the therapist's] not there and then during regular sessions he'll be getting other practices and you'll participate as well but the focus will be on him. And then, special instruction has a family component as well. Special instruction will be 30-minute sessions and then family training as well working with you and him, twice a month for 30 minutes. And as you heard earlier, this could be modified.”

When delivering this message, the official plainly released the above information from start to finish without any pause. Although her oral statement includes the proviso that it is a “recommendation,” the way it was conveyed seemed to imply that there was little room to wiggle within the recommendation. The presentation of the service recommendation seemed to express the view, “This is what will happen to you and your child.” The official did not ask if the recommendation met the parent's expectation, nor did the official ask if Lily agreed to the plan. Lily merely said “Okay,” and signed the paper to validate her agreement.

When reminiscing, Lily repeatedly mentioned her emotional strain in the course of meeting and interacting with many people in the process, especially in the first few months of the process after the referral when “her mind was so frazzled at that point.” Lily conveyed how interactions with early intervention personnel commonly occur with little care or understanding on the part of professionals regarding parents’ situations. Lily appealed to early intervention professionals:

Sometimes it seems as though the professionals who are involved with EI (instructors, therapists, administrators) don't really understand how difficult it is for parents to go through the EI process[es]. Perhaps it is unrealistic of me to expect them to know what it is like if they have never had a disabled child of their own. But … I have occasionally found myself frustrated by a lack of sensitivity about the realities of time constraints and psychological/emotional and financial resource limitations.

### Decision making throughout service provisioning process

Although parents’ active participation is safeguarded throughout the service provisioning process in the legislation, this right may not always be guaranteed, particularly due to lack of resources or knowledge (Bailey, Scarborough, & Hebbeler, 2003; Dunst, 2012; Mahoney & Filer, 1996; McWilliam et al., 1995; Summers et al., 2005). Likewise, Lily's experience demonstrated that decision making in the service provisioning process may not always make the family's voice a priority. For example, a physical therapist who came to evaluate Malcolm informed Lily of the results of the evaluation. Lily reflected:

The physical therapist said then that “I'm going to recommend that he receives physical therapy every week,” [And] she said, “I don't know [if he would qualify though].” She said to me that he is [on] borderline at that point. [So] she said [to] persuade the decision makers whoever that is. She said, “It is not my decision to make.”

This account implied that decision making does not belong to the parent or the evaluator. Lily then supplemented doctors’ letters about Malcolm's condition and their medical opinion to his file to ensure approval for services. At the IFSP meeting, followed by multiple evaluations, Lily asked, “We've been approved for physical therapy, right?” The official firmly countered, “No,” and clarified, “He hasn't been approved for any services. He was evaluated”; therefore, they were gathered to make that decision at the meeting. In addition, the official “asked Lily to be blunt so they know what they are dealing with and whatever his improvement was [as] it is not going to take away any services. [Then] Lily assured that she won't lie … [and] she won't be dishonest.” The scene almost resembled an interrogation of an offender in a court setting. The official seemed to warn Lily what to say and what not to say at the meeting. At the end of the meeting, it was determined that Lily's family was eligible for early intervention services. The family was accepted to receive physical therapy and special instruction in addition to family training in each discipline. To judge from the above incident, professionals seemed to place the parameters on “what is allowed” or “what happens next” in lieu of conveying the stance, “We all will work together in the process.”

In our interview, Lily discussed how parental rights in early intervention are viewed from her perspective: “The parents are granted many rights, but it seems as though it comes as a bit of a surprise when parents choose to exercise those rights—maybe most don't?” Lily showed doubt about the actual operation of family-centered practice, especially with respect to being conscious of parental rights and their value. This implies that parent participation, including decision making in early intervention, is required by law but, in real life, might be put into practice only in a limited way. The actual execution of the practice may vary despite how fully it is guaranteed on paper.

### The burden of the responsibility of family participation

Since the inception of early intervention, the components of “family-centered practice” and “active family participation” have been strengthened over the years. From the perspective of the parent, however, this approach can be viewed as unnecessary or overwhelming in families’ everyday lives. For example, Lily noted that it was very challenging scheduling with service providers. She reflected:

I find that scheduling is difficult so I have to take her [the therapist] when she's able to do it … because she's got a lot of other obligations and sometimes the times that she can do it aren't the most convenient times for us because I have to be doing something with H [Malcolm's older brother] or I'm taking Malcolm to medical appointments or whatever.

While plotting her course in the system of early intervention, she added,

It's overwhelming, truthfully, having so many people involved in our day-to-day life. They are all good, pleasant, knowledgeable people, but there are a lot of them! [In addition to] five therapists, he [Malcolm] has several medical professionals involved in his care. I often feel more like my son's case manager than his mom. It's a lot of organizing and coordinating.

She also ruminated how intrusive the process can be while maintaining relationships with various professionals.

It is odd to interact with therapists in our home so often and regularly, like we do with friends and family, but they are professionals with whom we have working relationships. It's a new type of relationship we have not experienced prior to our involvement with EI. We're neither coworkers, nor friends, and yet our relationships have aspects of both, e.g., we joke with one another, exchange information, share duties such as making phone calls to care providers, etc. At the same time, I feel it's important to maintain a level of professionalism, as I do not want to be inappropriate in being overly familiar with these people who are doing their jobs.

From this response, Lily brought out a critical thinking families in early intervention may have. For families, the numerous relationships they have to build can become an extra obligation in addition to caring for their young child. Of course, this challenge may vary by families’ individual experiences and their meaning; however, in Lily's world, it seemed that her interactions and partnerships were viewed as a burden that could intrude on the family's personal lives. In effect, policy makers’ view of “family-centered practice” can be differently construed by families.

## Discussion

The parent in this study conveyed her satisfaction with the actual services, especially regarding professionals’ knowledge and parental advocacy. At the same time, the parent shared frustration with the planning process and partnerships with providers. Lily described how she was overwhelmed by the number of people who came to be involved in her and her family's lives. She noted the providers’ lack of sensitivity about the realities the family of a child with special needs has. Taking into account the family's exceptional circumstances, Lily wished that more emotional and psychological support could be offered rather than simply supplying services to meet tangible needs.

These findings are consistent with other studies and deepen our understanding of early intervention through a closer examination of the parent's experience in several ways. With regard to family–professional partnerships and interactions, first, the study showed that the qualities identified as those that advance the relationship between families and professionals were consistently lacking (Summers et al., 2005). In particular, in the IFSP meeting process, the parent reflected that there was a limited level of partnership between the officials and the parent. The meeting process was documenting rather than discussing parental needs and concerns. The interaction between the two parties did not present the qualities the family had expected, therefore, a lack of the qualities mentioned in previous studies, such as trust (Vohs, 1998), respect (Zhang, Bennett, & Dahl, 1999; Ziviani et al., 2013), open communication (Coogle & Hanline, 2014; James & Chard, 2010; Wehman & Gilkerson, 1999), equality (Gallagher et al., 2004; Ziviani et al., 2013), etc., persisted. Then again, Lily added other qualities, such as emotional and psychological support from the officials, to the existing qualities listed in previous studies. Although some studies have highlighted the importance of care and support in the relationship between families and professionals (Brotherson et al., 2010; Wehman & Gilkerson, 1999), this has not been an extensive view. This study broadened what “supportive care” connotes from a parent's perspective.

Second, in the course of the decision-making process, the professionals took account of the parents’ voice only in a limited way, as suggested by previous studies (Bailey et al., 2003; Dunst, 2012; Summers et al., 2005). For example, Florian (1995), Mahoney and Filer (1996), and McWilliam et al. (1995) found that parental concerns and choices are not always taken into account due to available resources within a locality of their residency, regardless of parental preferences. What is different from previous research is that the present study claims that the limitation was due to the position of professionals rather than lack of resources. Professionals’ voice was more prioritized than that of the family especially in the IFSP meeting, and this affected the interpersonal dynamics of the service provisioning process. The officials’ authoritative position limited the level of a partnership with the family and subsequently led to a lack of sensitivity when attempting to create a culture of collaboration with the family. Thus, Lily raised the issue of how parental rights are required on paper but their execution is not guaranteed in practice. A few studies have discussed parental rights and their operation in practice (Belcher, Hairston-Fuller, & McFadden, 2011; Turnbull et al., 2007; Valle & Aponte, 2002). Thus, this study documented a limitation of the practice and challenged the notion of parental participation.

Finally, the study described a parent's experiences and perceptions concerning interactions and partnerships. From Lily's experience, parent participation activities, such as partnering with professionals, developing relationships, exchanging information in the service process, etc., did not actually focus on the family's concerns and, therefore, became a burden. Those obligations in the service provisioning process led Lily to regard the process as overwhelming for the family. This aspect of lived voice of the family regarding their participation has not been discussed in the previous studies. Most of the time, more interactive, intense collaborations and partnerships have been mandated within the preset rules of service providers. Thus, the study validates the need for individuality when working with families and throughout their multileveled, intersubjective engagements (Hollan, 2001).

## Limitations

The first limitation of the study was, given the time limit of the study, that data collection occurred during the early intervention entry and service delivery process within the first 6 months. Thus, the limited time of data collection may result in limiting the perceptions of the families’ overall interactions and experiences in early intervention and only conveying the families’ earliest impressions or accounts of early intervention. In addition, the family's participation level varied, and the selection process of the family was narrow. Although the father of the family was present at times throughout the data collection period, the researcher's interactions predominantly involved the mother. As such, the family's perceptions in this study may primarily reflect the mother's reflections. Moreover, the parent in the study was recruited through a parent group website. Thus, she was already an involved parent to some extent seeing that she had viewed the recruitment posting on the website and initiated contact for participation in the study. Thus, the selection process of the participant in this study may have resulted in limited or selective findings.

## Conclusions

As any phenomenological ethnographer would claim, this study does not intend to offer a generalized result but rather an indication of a creeping reification of a parent's experiences in the IFSP process while keeping human subjectivity in mind (Hollan, 2001). Thus, my hope was to describe the interactions the parent engaged in and developed through the consciousness and the lived meaning of her world (Schutz, 1967). Now that we are more conscious about parental individuality and consciousness (i.e., perception) in undergoing the service process (Butt, 2004), research in this area should be continued. A similar study with similar questions utilizing a comparable methodology could be conducted to further examine the complexity of the lived experience and to counterbalance the limitations of this study (Hollan, 2001). Or additional experiments combining different research approaches, such as across-case studies and analyses, could be conducted to validate the findings of this study (Erwin, Brotherson, & Summers, 2011; McWilliam, 2000), in particular, families’ burden of responsibility in family-centered practice, which is a new finding of this study. All these attempts could be a contribution for the development of improved policies for early intervention programs regarding “family-centered practice” and “parental participation.” Rather than traditional abstract views espoused by the lawmakers’ and professionals’ perspective, isolated from actual parental voices and experiences, individualizing the family's experiences is needed to support their choices and participation in caring for their child throughout the early intervention process.

## Footnotes

1 The use of term varies by country, for example, early intervention, early childhood intervention, early childhood special education, etc. This paper uses the term early intervention to refer to special education services for young children and their families.

2 The parent consented to sharing the excerpt.

3 The data used in this article were first collected by the author for her doctoral dissertation.
